# Nutrient Profiling and Child-Targeted Supermarket Foods: Assessing a “Made in Canada” Policy Approach

**DOI:** 10.3390/ijerph16040639

**Published:** 2019-02-21

**Authors:** Charlene Elliott, Natalie V. Scime

**Affiliations:** 1 Department of Communication, Media, and Film, University of Calgary, Calgary, AB T2N 1N4, Canada; 2 Department of Community Health Sciences, University of Calgary, Calgary, AB T2N 1N4, Canada; natalie.scime@ucalgary.ca

**Keywords:** children, food marketing, policy, nutrition, Canada, nutrient profiling

## Abstract

Marketing unhealthy food and beverages to children is a pervasive problem despite the negative impact it has on children’s taste preferences, eating habits and health. In an effort to mitigate this influence on Canadian children, Health Canada has developed a nutrient profile model with two options for national implementation. This study examined the application of Health Canada’s proposed model to 374 child-targeted supermarket products collected in Calgary, AB, Canada and compared this with two international nutrient profile models. Products were classified as permitted or not permitted for marketing to children using the Health Canada model (Option 1 and Option 2), the WHO Regional Office for Europe model, and the Pan-American Health Organization (PAHO) model. Results were summarized using descriptive statistics. Overall, Health Canada’s Option 1 was the most stringent, permitting only 2.7% of products to be marketed to children, followed by PAHO (7.0%), WHO (11.8%), and Health Canada’s Option 2 (28.6%). Across all models, six products (1.6%) were universally permitted, and nearly 60% of products were universally not permitted on the basis of nutritional quality. Such differences in classification have significant policy and health-related consequences, given that different foods will be framed as “acceptable” for marketing to children—and understood as more or less healthy—depending on the model used.

## 1. Introduction

In October 2016, Health Canada launched the Healthy Eating Strategy, with the goal of improving the food environment for Canadians [1]. Alongside its commitment to improve nutrition information and access to nutritious foods, the strategy pledged to protect children from the influence of unhealthy food and beverage marketing. Providing such protection is the very premise of Bill S-228, The Child Health Protection Act, which the Senate is expected to pass in 2019. Bill S-228 will prohibit the marketing of unhealthy food and beverages to Canadian children under age 13 to help curb childhood obesity and to promote child health. In preparation for the implementation of Bill S-228, Health Canada has been developing the precise criteria for defining unhealthy foods and determining child-directed marketing.

This move to restrict food marketing to children reflects a broader cultural wave—and evidence base—documenting the deleterious effects of food marketing on children’s taste preferences and dietary habits. Nearly a decade ago, the World Health Assembly called on its member states to adopt the WHO Set of Recommendations on the Marketing of Foods and Non-alcoholic Beverages to Children [2], subsequently providing a detailed framework for policy makers to implement the recommendations [3]. The WHO recommendations specifically suggest that countries should develop a nutrient profiling model to guide the restriction of food marketing to children, drawing from “existing legislation and policies, as appropriate” [3] (p. 14).

However, in a 2018 follow up report evaluating the implementation of these recommendations [4], the WHO lamented the “limited impact” of existing policies on reducing children’s exposure to unhealthy foods [4] (p. 5)—even as it underscored the “unequivocal evidence” pointing to the harmful effects of food marketing on children’s consumption and weight [4] (p. 5). Food promotion to children, the report reminds us, leads to a “cascade of effects” resulting in weight gain and diet-related disease (p. 9) and governments must be looked to as “the key stakeholders” in terms of providing leadership and developing policy in this area (p. 10). Implementing nutrient profile models to identify the foods that may (or may not) be marketed to children is framed as part of this leadership. While the research on nutrient profile model effectiveness (at improving child health) or validity for correctly identifying healthy foods is still in its infancy [4,5,6,7], the hypothesis supporting the use of such models is compelling: restricting the marketing of unhealthy foods reduces children’s dietary intake of, and preference for, such foods.

Nutrient profiling policy implementation and evaluation at the country-level continues to gain momentum. Nutrient profile models have proliferated in recent years: a 2016 systematic review identified 78 government-led models, 16 of which had applications for restricting marketing to children [6]. Chile has been lauded for its exemplary and comprehensive approach to regulating food marketing to children [8], and many other nations such as New Zealand [9] and South Africa [10], among others [4,11] are developing or adapting these models and assessing their feasibility and impact on local food landscapes—a logical initial step prior to jurisdictional roll-out.

The purpose of this study was to examine the impact that Health Canada’s forthcoming model would have on the food industry’s ability to market child-targeted foods currently available in the Canadian supermarket, and to describe how this “made in Canada” policy solution compares to two international nutrient profiling systems.

## 2. Methods

All child-targeted products were purchased from two grocery stores in Calgary, AB during the winter of 2017. The Real Canadian Superstore and Safeway were selected as data collection sites because they represent stores from Canada’s two leading retail grocery and food distributors (Loblaws Companies Ltd. and Sobeys Inc.). Stores were visited on multiple occasions to ensure all relevant products were collected. Following previous studies [12,13], child-targeted foods were defined and identified as products with one of the following characteristics: the word “child” or “kid” in the brand or product name (e.g., EnviroKidz cereals or CLIF Kid energy bars), appeals to fun or play on the package, links with children’s movies or television programs (i.e., character licensing), and child-friendly graphics or games. Since this study was interested in the nutritional profile of “regular” foods targeted at children, “junk foods” (such as candy, chocolate, potato chips and sugary sodas) were excluded from data collection. Trained graduate students photographed and coded each product for multiple variables, including, for the purposes of this article, nutritional information and ingredients.

Products were assessed in light of Health Canada’s nutrient profile model recommendations for determining whether foods should be allowed to be marketed to children (see Table 1) [14]. Following the WHO recommendations to draw from existing policies, Health Canada used definitions outlined in the Canadian Food Inspection Agency’s regulations (namely, the specific nutrient content claims requirement [15]) for the nutrient content claims for foods “low in” sodium, sugars and saturated fat. Health Canada proposed two options using a nutrient threshold approach. Option 1, the stricter threshold, permits the marketing of products that are “low in” saturated fat, total sugars and sodium (approximately 5% of the daily value). Option 2 is less strict and permits the marketing of products that contain approximately 15% of the daily value for these nutrients [16]. Both options consider the serving size and reference amount in assessing nutrient density, the latter of which represents the amount of food typically eaten at one sitting. Foods were assigned a product category according to the Health Canada Table of Reference Amounts for Food [17] to obtain reference amount values for each product and, in keeping with the model instructions, foods with a reference amount of 50 g or less (*n* = 203) such as cereals, cookies, and crackers were assessed using an amount of 50 g (see Table 1, footnote b).

Health Canada’s proposed nutrient profile model permits all “foundational foods” (i.e., vegetables, fruits, proteins and minimally processed whole grains) regardless of nutritional content, because they are core to a healthy diet. As such, we excluded these items (such as applesauce purees with no added sugars) when assessing the nutritional criteria under Options 1 and 2, but *included* them when providing the assessment in terms of the overall number of products that would be permitted to be marketed to children. Purees that listed juice concentrate as an ingredient, *even if added for colour*, were *not* eligible for exemption given that Health Canada views any inclusion of juice concentrate as an added free sugar.

To assess the strictness of Health Canada’s proposed recommendations for food marketing to children, we then evaluated the dataset according to two additional nutrient profiling models: the WHO Regional Office for Europe Model [18] and the Pan American Health Organization’s (PAHO) model [19], both of which also categorize foods and assess them in light of particular nutrient content thresholds (see Appendix A for a description of the WHO and PAHO model classification processes and criteria). For both the WHO and PAHO models, products are classified as unsuitable for marketing to children if they exceed particular nutrient thresholds for fat, sodium or free sugars.

Arithmetic calculations were used to derive nutrient density values for each product in the dataset, which were then compared against the nutrient criteria for each of the three models, separately. Frequencies and proportions were calculated to summarize the results. All analyses were performed using the statistical software package Stata v15 (StataCorp, College Station, TX, USA) [20].

## 3. Results

### 3.1. Child-Targeted Food Product Categories

In total, 374 child-targeted food products were purchased for analysis. Most of these products were dry goods (77.5%), such as cereals, cookies, granola bars, fruit snacks, and drinks. Refrigerated and frozen foods (including breakfast foods, popsicles, and ice cream) were the second most common food category (10.4%), followed by dairy (7.8%) and refrigerated or frozen meals (2.9%). Only two of the child-targeted items were produce, namely mini oranges and bagged salad, which were made appealing to children with cartoon stickers (in the case of the oranges) or packaging with cartoons from Disney Pixar’s *Cars 3* movie (in the case of the lettuce). These two items, along with six fruit and/or vegetable purees (out of 21 purees total) would be exempted from any restrictions on marketing to children: that is, they would be allowed to be marketed to children without an examination of the nutrient profile (see Table 2). Stated differently, only 2% of the products in the dataset were “foundational foods” considered core to a healthy diet. Following these exclusions, 366 products remained for assessment by Health Canada’s proposed model for defining unhealthy foods that would not be allowed to be marketed to children.

### 3.2. Food Product Suitability for Marketing to Children

Table 3 details the number of food products in each category permitted for marketing to children according to each Health Canada nutrient threshold option. The table also assesses the food products in light of the WHO and PAHO nutrient profile models (which is discussed below).

#### 3.2.1. Health Canada Model

According to the Health Canada model, only 2.7% of the products would be permitted to be marketed to children under Option 1 (the “low in” approach) compared to almost 30% of products (28.6%) under Option 2. Products permitted under Option 1 were the (previously mentioned) foundational foods, a single granola bar, and one package of cookies. Option 2, however, permitted nearly one-third (30%) of dry good products and 41% of refrigerated and frozen foods. Food sub-categories with high proportions of products allowed under Option 2 included 73.7% of frozen ices and popsicles; 42.4% of cereals, 40.4% of granola bars, snacks, and cereal bars; 39.1% of fruit snacks and applesauces; and 32.2% of cookies and biscuits. Various drinks (such as Kraft Kool Aid) were also permitted under Option 2. Dairy and meat products only had one milk product (one percent milk) and one chicken product (chicken nuggets) that would be permitted to be marketed to children under the more lenient Option 2. Using Health Canada criteria, no refrigerated meals (such as Lunchables) would be permitted to be marketed to children.

To better understand which specific nutrient threshold (or combination) was most likely to make a product ineligible for marketing to children using Health Canada’s criteria, we examined all products that were blocked under each option in light of the thresholds they exceeded (see Table 4). Of the 364 products *not* permitted under Option 1, 38.7% exceeded the sugar threshold and 22.5% exceeded the sugar and sodium thresholds. Of the 267 products *not* permitted under Option 2, over half (55.8%) exceeded the sugar threshold, 13.9% exceeded the fat and sugar threshold, and 13.5% exceeded the sodium threshold. No products exceeded all three fat, sugar, and sodium thresholds under Option 2, whereas 15.1% of products did so under Option 1. Total sugar content appears to the be the most common threshold exceeded, regardless of the option applied.

#### 3.2.2. WHO Model

According to the WHO model, 11.8% of products would be suitable for marketing to children (see Table 3). Twenty-three of these products were dry goods, comprised of pastas and soups and fruit snacks and applesauce. Just over half of the dairy products in our dataset were permissible (55%), including 100% of milk/yogurt-based drinks and 77.8% of yogurts. Similar to the Health Canada model, all produce products were suitable for marketing to children, whereas none of the refrigerated meals were. Based on initial food categorization, 49.2% of products would not be permitted *regardless of their nutrient content*; among these were fruit gushers (fruit snacks), fruit juice, and ice cream bars.

#### 3.2.3. PAHO Model

According to the PAHO model, two produce items, three fruit purees, three fruit juices, and one milk product were classified as unprocessed or minimally processed and, by default, permitted to be marketed. Following these exclusions, 365 products remained for assessment according to PAHO nutrient thresholds, of which 8.2% were classified as processed and 91.8% were classified as ultra-processed.

According to the PAHO model, 7.0% of food products were permitted for marketing to children. Suitable products were predominantly dry goods: 11 drinks, 9 fruit snacks or applesauces, and 3 granola/cereal bars and snacks. One milk product and all produce products were permitted. Refrigerated and frozen foods, meals, and meats were all deemed not suitable for marketing to children.

To further examine the nutritional quality of processed and ultra-processed food products according to the PAHO assessment, we classified the products that would not be suitable for marketing to children according to the specific nutrient threshold they exceeded (see Table 5). The majority of products (86.5%) were excessive in free sugars. Approximately one-third (36.2%) of products were excessive in sodium, 32.5% were excessive in saturated fat, and 28.7% excessive in total fat. One in four products (27.0%) was deemed excessive in three or more nutrients.

### 3.3. Product Suitability Across all Nutrient Profile Models

Overall, six products (1.6%) satisfied the nutritional requirements for marketing to children across the Health Canada (both options), WHO, and PAHO models. Suitable products included unsweetened applesauce and bagged lettuce. By contrast, 222 products (59.4%) were deemed not permitted for marketing to children based on nutritional quality criteria in all three nutrient profile models. These included 100% of refrigerated and frozen meals, cheeses, refrigerated cookies, ice cream products, puddings, dressings and sauces, and peanut butters and spreads, as well as 88.9% of crackers, 85.7% of frozen breakfast foods, 83.3% of milk products, 67.8% of cookies, 60.9% of fruit snacks/applesauces, 57.6% of cereals, and 55.3% of granola/cereal bars and snacks. The remaining 146 products (39.0%) received different classifications for suitability for marketing to children depending on the model used. For example, KinniKritters Animal Cookies were permitted under both Health Canada model options, but not permitted under the WHO or PAHO models. Iogo Nano Apple Blueberry Fruit Puree and Yogurt was permitted under Health Canada Option 2 and WHO, but not Option 1 and PAHO. Of the 146 products with different classifications, 89 were permitted under Health Canada Option 2 only and not the other models.

## 4. Discussion

Health Canada’s development of a national model for regulating food marketing to children is an important step toward harmonizing nutrient profiling efforts occurring at the provincial level [21]. Our study examined the application of a proposed pan-Canadian model to a comprehensive dataset of child-targeted supermarket products, and compared this with two well-known international nutrient profiling models. Overall, Health Canada’s Option 1 (“low in”) was the most stringent model allowing only 2.7% of products, followed by PAHO (7.0% of products), and WHO (11.8% of products). Health Canada’s Option 2 (“high in”) was the most lenient, permitting almost 3 of every 10 products (28.6%). Across all models, there was agreement in classification for nearly two-thirds of child-targeted products. Very few foods (1.6%) were universally permitted for marketing to children, all of which were produce or fruit/vegetable puree products. Moreover, 59.4% of products were universally classified as *not permitted* on the basis of nutritional quality.

Our findings suggest that the majority of supermarket foods aimed at children were of low nutritional quality regardless of the model used. For example, sugar was the most common threshold exceeded with roughly 40–80% of products surpassing the indicated amount in the Health Canada and PAHO models. This aligns with previous research documenting the poor nutritional value of supermarket foods in both Canada and the United States [12,13,22,23,24]. For instance, Elliott’s study of child-targeted supermarket foods in Canada reports that 89% of kids’ products could be classified as having poor nutritional quality due to high levels of sugar, fat and/or salt [5]; a follow up study reveals that 65% of child-oriented products specifically marketed as “better-for-you” were of poor nutritional quality [6]. In the US, Harris and colleagues classified 397 food products purchased between 2006 and 2008 with cross-promotions targeted at children and adolescents as either “healthy” or “unhealthy” based on the US Institute of Medicine’s Nutrition Standards for Foods in Schools guidelines (in particular, the standards for snack foods and foods sold during school meal programs) [23]. A mere 18.4% of these products are classified as healthy, and the authors observed that nutritional quality of products declined during the study period.

Only one other study (by Labonte et al. [25]) has examined how different nutrient profiling systems would impact the proportion and types of packaged foods and beverages that would be permitted to be marketed to children in Canada. However, the research was conducted prior to Health Canada developing its own policy approach, and therefore cannot assess how Health Canada’s proposed options compare to other major international profiling systems or how it would impact food marketing to children in the country. Labonte and colleagues also evaluated supermarket foods in Canada using different nutrient profiling systems, observing—as we now echo—the considerable differences in terms of what is allowed to be marketed to children depending on the model used. Moreover, Labonte et al. draw attention to the work by Scarborough et al. [26] and Ni et al. [9] in suggesting that “the various models evaluated were more consistent in identifying foods that would not be eligible for marketing than in identifying eligible foods” (p. 1477). In our study, all of the nutrient profile models identified the same six products as “foundational foods”; that is, the models were united in identifying 1.6% of foods as positive for consumption. However, when it came to more processed fare, the models diverged substantially—ranging from allowing 1 in 4 products to be marketed to children to allowing 1 in 50. Worth noting is that Health Canada’s options represent opposite ends of the continuum, the strictest *and* the most lenient, with the WHO and PAHO models falling in between. Such differences in classification have significant policy and health-related consequences, given that different foods will be framed as “acceptable” for marketing to children—and understood as more or less healthy—depending on the model used. While no firm decisions have yet been made, including on whether food packaging itself would be subject to restrictions (this is still under consideration), Health Canada has indicated that Option 1, the most strict model, is their preferred option, which would effectively block the marketing to children of 97% of products we examined. This would be a bold policy step in terms of reducing the power and exposure [2] of marketing messages on children.

Two considerations are worth noting when it comes to implementing either nutrient profiling option proposed by Health Canada. First is the relative complexity of assessing food products under the model. In our experience, the classification of foods into product categories in order to identify the appropriate reference amounts was not always intuitive [17]. For example, when classifying fruit snack products such as Betty Crocker Scooby Doo Fruit Flavoured Snacks, we originally deliberated between “S.1-Snacks” which includes chips, popcorn, grain and pulse-based snacks, and fruit-based snacks or “U.10-Fruit leather, bar or mini pieces, that may or may not contain vegetable ingredients”. After inquiring to Health Canada, we learned that the correct food category was in fact “U1-Candies, confectionaries, and chocolates”. Identifying reference amounts based on correct classification of food products is a crucial first step for use of Health Canada’s model. Future documentation for the model could include clarification on food products that are less straight-forward to classify.

The second consideration pertains to the classification of foods outside of a nutrient profiling approach; namely, considering how *consumers* understand and categorize food, and classify foods as “healthy” and “unhealthy”. We know that children understand and classify foods in broad terms [27]. The question arises: will banning the promotion of particular fruit snacks or particular applesauces to children while *allowing* the marketing of other fruit snacks and applesauces work to make a difference in children’s understanding of these foods as healthy or less healthy options? Under Health Canada’s stricter Option 1 approach, only one brand of animal cookies would be permitted to be marketed. The packaging of those cookies prominently features images of the animal-shaped cookies contained in its colourful box. Packages of the permitted animal cookies look very similar to the packages of animal cookies not permitted under the proposed legislation. Should one assume that children will see advertising for the permitted animal cookies and ask their parents to purchase animal cookies by KinniKritters rather than Barnum animal cookies, Presidents’ Choice animal cookies or cookies from some other brand? Similarly, will parents see these variously branded animal cookies differently? One possible outcome is that an advertisement for, say, KinniKritters animal cookies will simply work to promote animal cookies more generally, encouraging the understanding of fun, animal-shaped cookies as “good foods to eat”.

Of the various models assessed, Health Canada’s Option 1 allows for the fewest products to be marketed to children, and therefore appears to be most conducive to reducing the power and exposure of unhealthy marketing messages on children. Health Canada flags Option 1 as the preferred model, moreover, because it is deemed necessary to shield children from exceeding the daily maximum level of intakes for sodium, saturated fat, as recommended by the WHO. However, the critical issue of how *consumers* understand and classify food—as well as considering the confusion caused by marketing of “healthy” foods that are parity products of “unhealthy” foods (e.g., applesauces, animal cookies, etc.)—is not solved by any of these nutrient profiling models. The WHO Set of Recommendations on the Marketing of Foods and Non-alcoholic Beverages to Children aims to help protect children from the promotion of high-fat, sugar and/or salt foods, but it also emphasizes the need to provide an “enabling food environment—one that fosters and encourages healthy dietary choices” for children [3] (p. 4). In this case, marketing “healthy” animal cookies to children (for example) might cause confusion around cookies writ large, thus undermining efforts to foster healthy dietary habits in children. Restricting all food and beverage marketing to children would, in our view, be the best strategy for avoiding this problem. Given that this is not the current policy direction of Health Canada, a secondary recommendation is to make child-targeted food packaging subject to the regulations.

### Strengths and Limitations

This is the first study to analyze how a “made in Canada” policy solution for nutrient profiling would impact the foods currently marketed to children in the supermarket. Study strengths include analyzing a comprehensive dataset of child-targeted products, visiting the data collection sites multiple times to ensure all relevant products were captured, and data verification by multiple research assistants to ensure accuracy. While the product dataset is representative—products were collected from Canada’s two largest grocery retailers, and the same national brands are found across the country—adding products from smaller retailers would make it even more comprehensive.

## 5. Conclusions

This study provides a timely look at how Health Canada’s proposed nutrient profiling options would impact (in terms of being permitted for marketing to children) the child-targeted foods that are currently available in the Canadian supermarket. The research also reveals that the “made in Canada” policy solution bookends the other major nutrient profiling models designed by PAHO and WHO, by providing both the most stringent and the most lenient approaches to evaluating foods and beverages. All four options (Health Canada Option 1, PAHO, WHO and Health Canada Option 2) reveal that the nutritional quality of child-targeted foods in Canada is a problem: the four options harmonize on finding that nearly 60% of products would not be permitted for marketing to children, and in identifying sugar as a particular nutrient of concern. These findings prove unsettling given that the products in the dataset were selected because they explicitly targeted children or had packaging claiming that the product was for kids. When almost 60% of products designed for children would not be permitted to be marketed to them, there is a problem. However, the span between the number of products permitted across all four nutrient profiling options—from 1 in 50 products permitted to 1 in 4—revealed the substantial variation in terms of what is understood to be acceptable for marketing to our children. This pluralization of expertise, or what might be considered the fracturing of expert knowledge when it comes to nutrient profiling (between Health Canada, PAHO and WHO) comes with real consequences—in terms of what is understood as permissible for marketing to children, and by extension, healthy to eat.

## Figures and Tables

**Table 1:** Health Canada’s proposed nutrient profile model: Nutrient thresholds considered for restricting marketing to children.

Nutrient	Option 1 (“Low in” Nutrient Content Claim)		Option 2 (“High in” Nutrient Content Claim) ^a^	
	Foods ^b^ (~5% of the DV)	Prepackaged Meals and Combination Dishes ^c,d^	Foods ^b^ (15% of the DV)	Prepackaged Meals and Combination Dishes ^c,d^ (30% of the DV)
Saturated Fat (SFA)	≤2 g SFA + TFA per RA and ^e^ serving of stated size; and ≤15% energy from the sum of SFA + TFA ^f^	≤2 g SFA + TFA per 100 g; and ≤15% energy from the sum of SFA + TFA	<3 g per RA and per serving of stated size	<6 g per RA and per serving of stated size
Total Sugars	≤5 g per RA and per serving of stated size ^g^	≤5 g per 100 g	<15 g per RA and per serving of stated size	<30 g per RA and per serving of stated size
Sodium	≤140 mg per RA and serving of stated size ^h^	≤140 mg per 100 g	<345 mg per RA and per serving of stated size	<690 mg per RA and per serving of stated size

DV, daily value; RA, reference amount; SFA, saturated fatty acid; TFA, trans fatty acid. ^a^ Option 2 reflects the proposed nutrient thresholds for “high in” Front of Package (FOP) symbols [16]. ^b^ Reference amounts represent the amounts of food typically eaten at one sitting. Reference amounts can be found in the Table of Reference Amounts for Food [17], incorporated by reference into the Food and Drug Regulations. If the reference amount is 50 g (or 50 mL for liquids) or less, the food is assessed per 50 g or 50 mL (certain exceptions to this adjustment may apply, such as healthy oils with less than 30% of total fat as saturated and trans fat). ^c^ Inclusion of combination dishes is not in the original nutrient content claim but is proposed for inclusion in this context so that these types of foods can be assessed. ^d^ For meals with several discrete components (e.g., beverage, main, side, and dessert), the criteria must be met by ALL components of the meal. In this case, the criteria for “foods” will apply to each component unless the component is a combination dish (e.g., lasagna) in which case it will be assessed on per 100 g basis. ^e^ The “and” means that the claim must be met per RA and per serving of stated size in order for a product to be marketed to children. For example, a yogurt with a 175 g RA that is sold in a 100 g container (stated serving size), must meet the nutrient criteria for both amounts of food. ^f^ The threshold for saturated fat aligns with the “low in” nutrient content claim [15]. ^g^ The threshold for sugars is based on the “low in sugars” nutrient content claim proposed in the Front of Package consultation document [16]. ^h^ The thresholds align with “low in” nutrient content claims for sodium [15]. Table reproduced from a Health Canada discussion paper [14].

**Table 2:** Child-targeted food products classified as foundational foods according to Health Canada model (*n* = 8 out of 374 products).

Product Name	Product Type
President’s Choice Apple Mango Sweet Potato Squeeze Fruit Snacks	Fruit puree
Buddy Fruits (Mango, Passion and Banana)	Fruit puree
Compliments Super Squeeze (Apple, Blueberry, Raspberry and Beet)	Fruit puree
Cuties Seedless California Mandarins	Fruit
Dole Cars Classic Iceberg	Vegetable
Go Gourmet Squoosh (Squabbleberry)	Fruit puree
Mott’s Fruitsations Fruit Rockets (Unsweetened Apple)	Fruit puree
Mott’s Fruitsations Fruit Rockets (Unsweetened Strawberry-Kiwi)	Fruit puree

**Table 3:** Number of food products allowed to be marketed to children according to the Health Canada, WHO, and PAHO models (*n* = 374 products).

Category	Total *n*	Health Canada		WHO n (%)	PAHO n (%)
		Option 1 ^a^ n (%)	Option 2 ^b^ n (%)		
Dry Goods	290	8 (2.8%)	87 (30.0%)	23 (7.9%)	23 (7.9%)
Cereal	59	0 (0%)	25 (42.4%)	0 (0%)	0 (0%)
Cookies and Biscuits	59	1 (1.7%)	19 (32.2%)	0 (0%)	0 (0%)
Crackers	18	0 (0%)	2 (11.1%)	0 (0%)	0 (0%)
Drinks and Drink Boxes	29	0 (0%)	4 (13.8%)	0 (0%)	11 (37.9%)
Dressings, Sauces, Condiments	4	0 (0%)	0 (0%)	0 (0%)	0 (0%)
Fruit Snacks and Applesauce*	46	6 (13.0%)	18 (39.1%)	8 (17.4%)	9 (19.6%)
Granola/Cereal Bars and Snacks	47	1 (2.1%)	19 (40.4%)	0 (0%)	3 (6.4%)
Pasta (Boxed/Canned) and Soups	25	0 (0%)	0 (0%)	15 (60.0%)	0 (0%)
Peanut Butters and Spreads	1	0 (0%)	0 (0%)	0 (0%)	0 (0%)
Puddings and Jell-O’s	2	0 (0%)	0 (0%)	0 (0%)	0 (0%)
Dairy	29	0 (0%)	1 (3.4%)	16 (55.2%)	1 (3.4%)
Cheese	6	0 (0%)	0 (0%)	0 (0%)	0 (0%)
Milk	6	0 (0%)	1 (16.7%)	1 (16.7%)	1 (16.7%)
Milk/Yogurt-based Drinks	8	0 (0%)	0 (0%)	8 (100%)	0 (0%)
Yogurt	9	0 (0%)	0 (0%)	7 (77.8%)	0 (0%)
Produce	2	2 (100%)	2 (100%)	2 (100%)	2 (100%)
Fruit *	1	1 (100%)	1 (100%)	1 (100%)	1 (100%)
Vegetable *	1	1 (100%)	1 (100%)	1 (100%)	1 (100%)
Refrigerated/Frozen Foods	39	0 (0%)	16 (41.0%)	0 (0%)	0 (0%)
Fries and Potatoes	1	0 (0%)	1 (100.0%)	0 (0%)	0 (0%)
Frozen Breakfast Foods	7	0 (0%)	1 (14.3%)	0 (0%)	0 (0%)
Frozen Ices and Popsicles	19	0 (0%)	14 (73.7%)	0 (0%)	0 (0%)
Ice Cream	9	0 (0%)	0 (0%)	0 (0%)	0 (0%)
Refrigerated Cookies	3	0 (0%)	0 (0%)	0 (0%)	0 (0%)
Refrigerated/Frozen Meal	11	0 (0%)	0 (0%)	0 (0%)	0 (0%)
Packaged Lunch	10	0 (0%)	0 (0%)	0 (0%)	0 (0%)
Pizza Pops and Pogos	1	0 (0%)	0 (0%)	0 (0%)	0 (0%)
Meat	3	0 (0%)	1 (33.3%)	3 (100%)	0 (0%)
Chicken	2	0 (0%)	1 (50.0%)	2 (100%)	0 (0%)
Fish	1	0 (0%)	0 (0%)	1 (100%)	0 (0%)
Overall	374	10 (2.7%)	107 (28.6%)	44 (11.8%)	26 (7.0%)

WHO, World Health Organization; PAHO, Pan-American Health Organization. * Sub-category includes foundational foods according to Health Canada Model. All fruits and vegetables are foundational foods, and six applesauce (which includes fruit puree) products are foundational foods. ^a^ “Low In” criteria (~5% DV). ^b^ “High in” criteria (15% DV).

**Table 4:** Proportion of food products that are not permitted to be marketed to children according to specific nutrient threshold(s) exceeded in the Health Canada model.

Nutrient Threshold(s)	Option 1: “Low In” (*n* = 364)		Option 2: “High In” (*n* = 267)	
	*n*	(%)	*n*	(%)
Saturated Fat	0	(0%)	19	(7.1%)
Total Sugars	141	(38.7%)	149	(55.8%)
Sodium	27	(7.4%)	36	(13.5%)
Saturated Fat and Total Sugars	43	(11.8%)	37	(13.9%)
Saturated Fat and Sodium	16	(4.4%)	20	(7.5%)
Total Sugars and Sodium	82	(22.5%)	6	(2.2%)
Saturated Fat and Total Sugars and Sodium	55	(15.1%)	0	(0%)

**Table 5:** Proportion of processed and ultra-processed food products that are not permitted to be marketed to children according to which specific nutrient threshold(s) they exceeded in the PAHO model (*n* = 348).

PAHO Nutrient Threshold Criteria	*n*	(%)
Excessive in total fat	100	28.7%
Excessive in saturated fat	113	32.5%
Excessive in trans fat	16	4.6%
Excessive in sodium	126	36.2%
Excessive in free sugars	301	86.5%
Excessive in other sweeteners	41	11.8%
Excessive in 1 of the above	129	37.1%
Excessive in 2 of the above	125	35.9%
Excessive in 3 or more of the above	94	27.0%

## Appendix A

### Description of Processes and Criteria for WHO and PAHO Nutrient Profile Models

#### WHO Regional Office for Europe Model [18]

Food products are first categorized according to the type of food:

1. Chocolate and sugar confectionary (including energy bars, sweet topping, and desserts)
2. Cakes, cookies and other sweet baked goods
3. Savoury snacks
4. Beverages: (a) Juices; (b) Milk drinks; (c) Energy drink; (d) Other beverages
5. Edible ices
6. Breakfast cereals
7. Yogurt, sour milk, cream and similar foods
8. Cheese
9. Ready-made and convenience foods
10. Butter and other fats and oils
11. Bread and bread products
12. Fresh or dried pasta, rice, and grains
13. Fresh and frozen meat, poultry, and fish
14. Processed meat, poultry, and fish
15. Fresh and frozen fruit, vegetables, and legumes
16. Processed fruit, vegetables, and legumes
17. Sauces, dips, and dressings

Products classified into one of chocolate and confectionery; cakes, cookies and other sweet baked goods; juices; energy drinks; and edible ices are *not* permitted regardless of their nutrient content. Products in the remaining categories are then assessed based on category-specific nutrient content thresholds per 100 g/mL servings for total fat, saturated fat, total sugar, added sugar, non-sugar sweetener, salt, and energy (see Annex 1 of the WHO Regional Office for Europe Nutrient Profile Model Report [18] for a detailed description of nutrient criteria per food category).

#### PAHO Model [19]

Food products are first categorized according to level of processing:

1. Unprocessed/minimally processed
2. Processed
3. Ultra-processed

Unprocessed/minimally processed foods are permitted regardless of their nutrition content. Processed and ultra-processed foods are then assessed as to whether they are excessive in:

- Total fat: ≥30% of total energy from fat
- Saturated fat: ≥10% of total energy
- Trans fat: ≥1% of total energy
- Sodium: ≥1 mg per 1 kcal
- Free sugar: ≥10% of total energy; estimated using the total sugar content declared on nutrition labels and formulas suggested by PAHO
- Other sweeteners: present in any amount

## References

[1] Health Canada Health Canada’s Healthy Eating Strategy. https://www.canada.ca/en/services/health/campaigns/vision-healthy-canada/healthy-eating.html#a4.

[2] World Heath Organization Set of Recommendations on the Marketing of Foods and Non-Alcoholic Beverages to Children. http://apps.who.int/iris/bitstream/handle/10665/44416/9789241500210_eng.pdf;jsessionid=4DA284276CF8B522B7F886DBDF6776D8?sequence=1.

[3] World Heath Organization A Framework for Implementing the Set of Recommendations on the Marketing of Foods and Non-Alcoholic Beverages to Children. http://apps.who.int/iris/bitstream/handle/10665/80148/9789241503242_eng.pdf?sequence=1.

[4] World Heath Organization Evaluating Implementation of the WHO Set of Recommendations on the Marketing of Foods and Non-Alcoholic Beverages to Children: Progress, Challenges and Guidance for Next Steps in the WHO European Region. http://www.euro.who.int/__data/assets/pdf_file/0003/384015/food-marketing-kids-eng.pdf.

[5] Rayner M., Scarborough P., Kaur A. (2013). Nutrient profiling and the regulation of marketing to children. Possibilities and pitfalls. Appetite.

[6] Labonté M.È., Poon T., Gladanac B., Ahmed M., Franco-Arellano B., Rayner M., L’Abbé M.R. (2018). Nutrient Profile Models with Applications in Government-Led Nutrition Policies Aimed at Health Promotion and Noncommunicable Disease Prevention: A Systematic Review. Adv. Nutr..

[7] Poon T., Labonté M.È., Mulligan C., Ahmed M., Dickinson K.M., L’Abbé M.R. (2018). Comparison of nutrient profiling models for assessing the nutritional quality of foods: A validation study. Br. J. Nutr..

[8] Corvalán C., Reyes M., Garmendia M.L., Uauy R. (2019). Structural responses to the obesity and non-communicable diseases epidemic: Update on the Chilean law of food labelling and advertising. Obes. Rev..

[9] Mhurchu C.N., Mackenzie T., Vandevijvere S. (2016). Protecting New Zealand children from exposure to the marketing of unhealthy foods and drinks: A comparison of three nutrient profiling systems to classify foods. N. Z. Med. J..

[10] Wicks M., Wright H., Wentzel-viljoen E. (2017). Restricting the marketing of foods and non-alcoholic beverages to children in South Africa: are all nutrient profiling models the same?. Br. J. Nutr..

[11] Vandevijvere S., Barquera S., Caceres G., Corvalan C., Karupaiah T., Kroker-Lobos M.F., L’Abbé M., Ng S.H., Phulkerd S., Ramirez-Zea M. (2019). An 11-country study to benchmark the implementation of recommended nutrition policies by national governments using the Healthy Food Environment Policy Index, 2015–2018. Obes. Rev..

[12] Elliott C. (2012). Packaging Health: Examining “Better-for-You” Foods Targeted at Children. Can. Public Policy.

[13] Elliott C. (2008). Assessing “fun foods”: Nutritional content and analysis of supermarket foods targeted at children. Obes. Rev..

[14] Health Canada toward Restricting Unhealthy Food and Beverage Marketing to Children: Discussion Paper for Public Consultation. https://www.healthyeatingconsultations.ca/discussion-paper.

[15] Canadian Food Inspection Agency Specific Nutrient Content Claim Requirements. http://www.inspection.gc.ca/food/general-food-requirements-and-guidance/labelling/for-industry/nutrient-content/specific-claim-requirements/eng/1389907770176/1389907817577.

[16] Health Canada Towards Front-of-Package Nutrition Labels for Canadians. https://www.canada.ca/content/dam/canada/health-canada/migration/health-system-systeme-sante/consultations/labels-nutrition-etiquetage/alt/labels-nutrition-etiquetage-eng.pdf.

[17] Health Canada Nutrition Labelling—Table of Reference Amounts for Food. https://www.canada.ca/en/health-canada/services/technical-documents-labelling-requirements/table-reference-amounts-food/nutrition-labelling.html#m..

[18] World Health Organization WHO Regional Office for Europe Nutrient Profile Model. http://www.euro.who.int/__data/assets/pdf_file/0005/270716/Nutrient-children_web-new.pdf?ua=1.

[19] Pan American Health Organization Pan American Health Organization Nutrient Profile Model. http://iris.paho.org/xmlui/bitstream/handle/123456789/18621/9789275118733_eng.pdf?sequence=9&isAllowed=y.

[20] Statacorp (2017). Stata Statistical Software: Release 15.

[21] Olstad D.L., Poirier K., Naylor P.J., Shearer C., Kirk S.F.L. (2015). Policy outcomes of applying different nutrient profiling systems in recreational sports settings: The case for national harmonization in Canada. Public Health Nutr..

[22] Federal Trade Commission Marketing Food to Children and Adolescents: A Review of Industry Expenditures, Activities, and Self-Regulation. https://www.ftc.gov/sites/default/files/documents/reports/marketing-food-children-and-adolescents-review-industry-expenditures-activities-and-self-regulation/p064504foodmktingreport.pdf.

[23] Harris J.L., Schwartz M.B., Brownell K.D. (2010). Marketing foods to children and adolescents: Licensed characters and other promotions on packaged foods in the supermarket. Public Health Nutr..

[24] Lapierre M.A., Brown A.M., Houtzer H.V., Thomas T.J. (2017). Child-directed and nutrition-focused marketing cues on food packaging: Links to nutritional content. Public Health Nutr..

[25] Labonté M.È., Poon T., Mulligan C., Bernstein J.T., Franco-Arellano B., L’Abbé M.R. (2017). Comparison of global nutrient profiling systems for restricting the commercial marketing of foods and beverages of low nutritional quality to children in Canada. Am. J. Clin. Nutr..

[26] Scarborough P., Payne C., Agu C.G., Kaur A., Mizdrak A., Rayner M., Halford J.C.G., Boyland E. (2013). How important is the choice of the nutrient profile model used to regulate broadcast advertising of foods to children: A comparison using a targeted data set. Eur. J. Clin. Nutr..

[27] Elliott C. (2011). “It’s junk food and chicken nuggets”: Children’s perspectives on ‘kids’ food’ and the question of food classification. J. Consum. Behav..

